# Effects of intravenous l-carnitine on myocardial fatty acid imaging in hemodialysis patients: responders or non-responders to l-carnitine

**DOI:** 10.1186/s40064-015-1119-z

**Published:** 2015-07-16

**Authors:** Masato Nishimura, Toshiko Tokoro, Toru Takatani, Nodoka Sato, Masaya Nishida, Tetsuya Hashimoto, Satoru Yamazaki, Hiroyuki Kobayashi, Toshihiko Ono

**Affiliations:** Cardiovascular Division, Toujinkai Hospital, 83-1, Iga, Momoyama-cho, Fushimi-ku, Kyoto, 612-8026 Japan; Department of Nephrology, Toujinkai Hospital, Kyoto, Japan; Department of Urology, Toujinkai Hospital, Kyoto, Japan; Department of Urology, Toujinkai Clinic, Kyoto, Japan

**Keywords:** Carnitine, Fatty acid, Heart, Hypoalbuminemia, Imaging, Uremic cardiomyopathy

## Abstract

We investigated whether chronic intravenous administration of l-carnitine could improve myocardial fatty acid imaging in patients on maintenance hemodialysis. We enrolled 72 hemodialysis patients who had impaired myocardial fatty acid imaging and left ventricular dysfunction not based on coronary lesion. l-Carnitine (1,000 mg) was intravenously administered after dialysis for 1 year to 36 participants (Carnitine group), while not in the other 36 participants (Control group). Single-photon emission computed tomography (SPECT) using an iodinated fatty acid analogue, BMIPP, was performed. Uptake on SPECT images was graded in 17 segments on a five-point scale (0, normal; 4, absent) and assessed as BMIPP summed scores. During follow-up, 19 participants were discontinued from the study, and 53 participants (65 ± 12 years: 27 carnitine, 26 control) were analyzed. The mean BMIPP summed scores 1 year after carnitine administration did not differ from that before in the carnitine group, nor from that in the control group. However, improved SPECT (Changes in BMIPP summed scores <−20%) was found in 7 (25.9%) participants in the carnitine, whereas in 2 (7.7%) in the control group. Multivariate logistic analysis showed the improved SPECT was inversely associated with baseline serum albumin levels (1 g/L: odds ratio, 0.669); the cut-off was 35 g/L. Chronic intravenous l-carnitine might improve myocardial fatty acid imaging in a selected group of hemodialysis patients with hypoalbuminemia.

## Background

Carnitine plays an important role in myocardial fatty acid metabolism by transporting long-chain free fatty acids (FFA) from the cytoplasm to the matrix of myocardial and skeletal muscle mitochondria for β-oxidation. The effects of l-carnitine on the cardiovascular complications of dialysis patients are still controversial, although supplementation of l-carnitine in dialysis patients has been reported to improve left ventricular (LV) dysfunction and arrhythmia in some studies (Van ES et al. 1992; Matsumoto et al. 2000; Romagnoli et al. 2002; Suzuki et al. 1982). Sakurabayashi et al. reported that chronic oral administration of l-carnitine to hemodialysis patients did not change myocardial accumulation of ^123^I-*β*-methyliodophenyl pentadecanoic acid (BMIPP), an iodinated analogue of free FFA, or LV dimension or function, but it increased the washout rate of ^123^I-BMIPP (Sakurabayashi et al. 1999). However, no study has been reported regarding the effect of l-carnitine on single-photon emission computed tomography (SPECT) using ^123^I-BMIPP, of which improvement may contribute to betterment of LV dysfunction or decrease in cardiac death (Nishimura et al. 2006, 2008a, b, 2011, 2014, 2015; Moroi et al. 2013). In the present study, we investigated whether chronic intravenous administration of l-carnitine could improve impaired myocardial fatty acid imaging in patients on maintenance hemodialysis with LV dysfunction not based on obstructive coronary artery disease (CAD) or valvular heart diseases.

## Methods

### Study population

**Figure 1:**
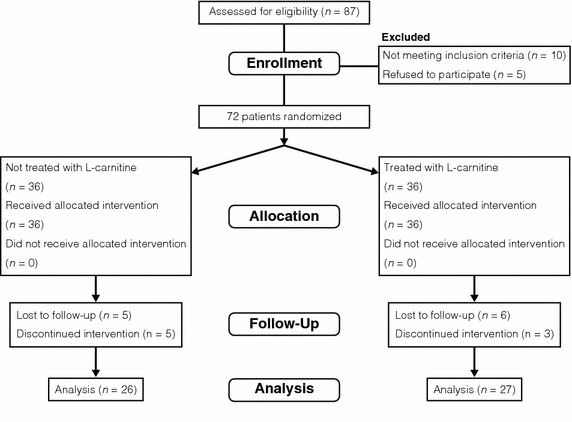
Participant flow chart.

Figure 1 shows a participant flow chart, which proceeded at the two dialysis centers associated with the Toujinkai Group: Toujinkai Hospital and Toujinkai Clinic. Eligibility criteria of this study were as follows: (1) Patients on chronic hemodialysis with a history of heart failure needing hospitalization (grade IVof New York Heart Association) except fluid overload from June 1st, 2012 to May 31st, 2013; (2) No significant obstructive CAD identified by angiography within one year of the study (from June 1st, 2012 to May 31st, 2013); (3) LV dysfunction evaluated by echocardiography: mildly or moderately reduced LV systolic function [left ventricular ejection fraction (LVEF) <55%] and/or LV hypertrophy, which indicates LV remodeling and lowered LV diastolic function; and (4) BMIPP summed scores (SS) ≧4, which was based on the results of the B-SAFE study (Moroi et al. 2013). Criteria for exclusion from participation were (1) Moderate or worse valvular heart disease; (2) Past history of acute or old myocardial infarction and/or coronary revascularization by percutaneous coronary intervention or coronary artery bypass grafting. Eighty-seven hemodialysis patients in the Toujinkai Group met the eligibility criteria; however, ten patients were excluded based on the exclusion criteria, and five patients refused to participate this study. Consequently, 72 hemodialysis patients were enrolled in this study between June 1st and 30th of 2013 (40 men and 32 women, mean age: 64 ± 11 years; mean dialysis duration: 146 ± 96 months). Simple randomization was performed by assigning the participants to the carnitine or control group (1:1) in the order of enrollment; the person who was not involved in this study performed this randomized assignment of participants to the two groups. From August 1st, 2013 to July 31st, 2014, l-carnitine (l-Cartin^®^ FF, Otsuka Pharmaceutical Co., Ltd. Tokyo, Japan) was intravenously administered after each dialysis session to 36 participants (Carnitine group), while the other 36 participants were not administered l-carnitine (Control group). The dose of intravenous administration of l-carnitine was uniformly 1,000 mg on each hemodialysis session. The Ethics Committee for Human Research of the Toujinkai Group approved the study protocol, and all participants provided written, informed consent to participate in all procedures associated with the study. The study was performed in accordance with the principles of the Declaration of Helsinki, and registered to the *ClinicalTrials.gov* (https://www.clinicaltrials.gov/): protocol identifier, NCT02322697.

### Coronary angiography (CAG)

Experienced interventional cardiologists performed quantitative CAG at the Department of Interventional Cardiology of Kyoto Second Red Cross Hospital using a validated automated edge-detection program (CCIP-310/W, CATHEX, Tokyo, Japan). Significant coronary artery stenosis was defined as stenosis of >50% diameter on CAG images.

### Radionuclide imaging

All participants underwent resting ^123^I-BMIPP SPECT after fasting for over 6 h on a midweek, non-dialysis day within 1 month before the study and at 1 year after starting the study. Details of the dual BMIPP SPECT procedure are described elsewhere (Nishimura et al. 2006, 2008a, b, 2011). The images were divided into 17 segments for semiquantitative analysis according to the standard myocardial segmentation for tomographic heart imaging established by the American Heart Association. The amount of radioactivity taken up by each segment was visually graded and assigned an uptake score of 0 (normal), 1 (mildly reduced), 2 (moderately reduced), 3 (severely reduced), or 4 (none). The BMIPP SPECT scores for 17 myocardial segments were designated as BMIPP SS. The same experienced technician performed all scintigraphic procedures. All BMIPP SPECT images were interpreted within one week of the SPECT examination by the same two investigators. Both of them interpreted SPECT images at the same time without knowledge of the identity (name), clinical condition (age, gender, blood pressure, presence or absence of diabetes mellitus, cardiothoracic ratio, dialysis duration and cardiac functions evaluated by echocardiography), and laboratory data about the participant. The information about the assignment of participants to carnitine or control group was not given to these two investigators at the interpretation of BMIPP SPECT.

### Echocardiography

The participants underwent two-dimensionally guided echocardiography using a single ultrasonographic recorder (HD11XD, Philips, the Netherlands) on a midweek non-dialysis day within 1 month before the study, 6 months, and 12 months after starting the study. LVEF levels were quantified using the biplanar Simpson’s rule, and left ventricular mass was measured as recommended by the American Society of Echocardiography. Mitral early to atrial (E/A) wave velocity ratio was measured as an index of LV diastolic function. Left ventricular mass was normalized to body surface area, and is described herein as left ventricular mass index (LVMI). Criteria for LV hypertrophy were an LVMI exceeding 134 g/m^2^ in men or 110 g/m^2^ in women (Sahn et al. 1978).

### Biochemical and hematological determinations

On the first hemodialysis session of the week within 30 days before starting the study, blood samples (10 ml) were obtained from patients who had fasted overnight and rested for 10 min. Blood hemoglobin (Hb), plasma B-type natriuretic peptide (BNP) concentration, and serum concentrations of calcium, inorganic phosphorus, albumin, total cholesterol, C-reactive protein (CRP), and intact parathyroid hormone were determined. Plasma BNP concentrations were measured additionally at 6 months and 12 months after starting the study. We used fasting plasma glucose and fasting plasma insulin concentrations to calculate the homeostasis model assessment of insulin resistance (HOMA-IR) as fasting glucose concentration (mmol/L) × fasting insulin concentration (μU/ml)/22.5. Blood samples were collected on the same day to measure this and other biochemical and hematological parameters. Serum concentrations of total, free, and acyl carnitine were determined within 30 days before the study, and 3, 6, and 12 months after starting the study using enzymatic cycling method (SRL, Inc. Tokyo, Japan) (Takahashi et al. 1994). The same erythropoiesis stimulating agent (ESA) (Epoetin beta pegol, C.E.R.A., Chugai Pharmaceutical Co., Ltd., Tokyo, Japan) was administered to all participants. The ESA resistance index (ERI) was determined as the monthly weight-adjusted dose of ESA (μg/kg) divided by Hb concentration (g/L). Changes in ERI before and 1 year after carnitine administration were calculated as follows: (ERI at 1 year after carnitine administration–ERI before carnitine administration)/ERI before carnitine administration × 100 (%). We used the dose of ESA and mean blood Hb of the month just before starting this study (July, 2013) for calculation of ERI before carnitine administration and the dose of ESA and mean blood Hb of the last month of this study (July, 2014) for calculation of ERI after carnitine administration. Decrease in ERI over 50% was defined as improvement of ERI, whereas increase in ERI over 50% as aggravation of ERI.

### Statistical analysis

Values are expressed as mean ± SD. We compared the means of continuous variables using paired or non-paired *t* tests. Categorical data were analyzed using the χ^2^ test. Threshold for the serum albumin concentration for changes in BMIPP SS <−20% was defined using receiver-operating characteristic (ROC) analysis. Any covariates that tended to be significant in univariate logistic analyses (*P* < 0.1) were assessed by multiple logistic analysis. *P* values <0.05 were considered significant. Individuals without knowledge of the participants’ profiles and clinical data performed all statistical analyses. All statistical analyses were performed with SAS software version 8.2.

## Results

During the follow-up of 72 participants from Aug 1st, 2013 to July 31st, 2014, 11 participants were lost for follow-up, and 8 participants were discontinued from this study (Figure 1). In the carnitine group (*n* = 36), 5 participants died (2 sudden death, 2 infection, 1 heart failure), one participant changed the dialysis center, and three participants dropped out from the study (one participant had severe diarrhea after administering l-carnitine, and the other two participants had refused subsequent BMIPP SPECT). In the control group (*n* = 36), 5 participants died (2 sudden death, 2 heart failure, 1 malignancy), and five participants had refused subsequent BMIPP SPECT. Consequently, we analyzed the data of 53 participants (27 men and 26 women; mean age, 65 ± 12 years; hemodialysis duration, 149 ± 105 months: carnitine group, *n* = 27; control group, *n* = 26). Clinical baseline characteristics did not differ between the carnitine and control groups (Table 1).

**Table 1:** Baseline clinical characteristics in the control and cartinine groups

	Control (*n* = 26)	Carnitine (*n* = 27)	*P*
Age, y	64.3 ± 12.9	64.7 ± 12.0	0.892
Male gender, *n* (%)	14 (53.9)	14 (51.9)	0.887
Dialysis duration, months	141.2 ± 83.8	149.1 ± 105.2	0.763
Smoking, *n* (%)	9 (34.6)	9 (33.3)	0.923
Alcohol, *n* (%)	8 (30.8)	8 (29.6)	0.930
Diabetes mellitus, *n* (%)	13 (50.0)	14 (51.9)	0.895
Systolic blood pressure before dialysis, mm Hg	138.0 ± 12.1	134.0 ± 18.8	0.360
Diastolic blood pressure before dialysis, mm Hg	73.4 ± 10.6	70.5 ± 14.7	0.408
Body mass index, kg/m^2^	21.2 ± 4.5	22.9 ± 4.1	0.170
Cardiothoracic ratio, %	53.1 ± 5.8	53.3 ± 4.9	0.922
Left ventricular ejection fraction, %	52.2 ± 11.9	53.2 ± 11.9	0.696
Left ventricular mass index, g/m^2^	126.6 ± 24.8	126.4 ± 24.1	0.962
Mitral early to atrial (E/A) wave velocity ratio	1.2 ± 0.6	1.1 ± 0.6	0.875
Blood hemoglobin, g/L	106.8 ± 10.8	106.1 ± 9.0	0.800
Serum albumin, g/L	37.8 ± 2.9	37.9 ± 3.4	0892
Serum calcium, mmol/L	2.2 ± 0.1	2.2 ± 0.1	0.451
Serum inorganic phosphorus, mmol/L	1.6 ± 0.4	1.6 ± 0.3	0.810
Serum total cholesterol, mmol/L	4.1 ± 0.6	3.9 ± 1.1	0.515
Serum ferritin, pmol/L	273.9 ± 185.8	281.8 ± 197.7	0.859
Serum intact parathyroid hormone, ng/L	176.1 ± 99.3	133.4 ± 106.0	0.136
Serum C-reactive protein, mg/L	2.5 ± 2.2	2.2 ± 2.6	0.635
Plasma B-type natriuretic peptide, ng/L	291.2 ± 174.6	249.2 ± 229.1	0.457
HOMA-IR, mmol/L・μU/ml	5.9 ± 1.9	5.3 ± 2.3	0.294
BMIPP summed score	18.2 ± 6.0	18.9 ± 11.3	0.794
Medications			
*α*_1_ blockers, *n* (%)	2 (7.7)	3 (11.1)	0.677
*β* blockers, *n* (%)	17 (65.4)	17 (63.0)	0.858
Calcium blockers, *n* (%)	7 (26.9)	7 (25.9)	0.936
RAS inhibitors, *n* (%)	8 (30.8)	9 (33.3)	0.845
Nitrates, *n* (%)	3 (11.5)	3 (11.1)	0.962
Antiplatelet drugs, *n* (%)	16 (61.5)	18 (66.7)	0.704
Anticoagulation drugs, *n* (%)	3 (11.5)	5 (18.5)	0.487
Statins, *n* (%)	7 (26.9)	7 (25.9)	0.936

*HOMA-IR* the homeostasis model assessment index of insulin resistance, *RAS* renin-angiotensin system.

### Circulating carnitine and BNP concentrations and cardiac functions

Compared with the normal ranges of serum carnitine concentrations in the laboratory used for measurement in this study (total carnitine, 45–91 μmol/L; free carnitine, 36–74 μmol/L; acyl carnitine, 16–23 μmol/L) (Takahashi et al. 1994), mean baseline serum concentration of free carnitine seemed to be lower, and that of acyl carnitine higher in the participants. Mean baseline serum concentration of total carnitine did not differ with that of normal controls (Table 2). Mean serum concentrations of total, free, and acyl carnitine increased at 3 months of intravenous administration of l-carnitine. Mean serum concentrations of total and free carnitine were further increased at 12 months compared with 3 or 6 months of carnitine administration, whereas mean serum concentration of acyl carnitine did not differ among 3, 6, or 12 months of carnitine administration. Mean values of acyl/free carnitine ratio did not differ at 3, 6, or 12 months of carnitine administration compared with before administration, but decreased at 12 months compared with 3 or 6 months (Table 2).

**Table 2:** Changes in serum concentrations of carnitine after intravenous administratin of l-carnitine

	Before	3 months	6 months	12 months
Total carnitine, μmol/L	62.4 ± 59.0	380.8 ± 96.0^*^	406.6 ± 73.3^*^	441.1 ± 101.9^*††∫^
Free carnitine, μmol/L	37.6 ± 38.3	220.9 ± 52.7^*^	236.4 ± 41.5^*^	264.9 ± 59.2^*††∬^
Acyl carnitine, μmol/L	24.7 ± 21.3	159.9 ± 49.1^*^	170.2 ± 43.8^*^	176.1 ± 51.5^*^
Acyl/free carnitine ratio	0.70 ± 0.14	0.72 ± 0.14	0.73 ± 0.17	0.67 ± 0.1^†∫^

^*^ *P* < 0.01 versus before; ^†^ *P* < 0.05 versus 3 months; ^††^ *P* < 0.01 versus 3 months; ^∫^ *P* < 0.05 versus 6 months; ^∬^ *P* < 0.01 versus 6 months.

In the control group, mean values of plasma BNP concentration, LVEF, and LVMI did not differ among before carnitine administration, at 6 months, and at 12 months of administration (Table 3). In the carnitine group, mean values of plasma BNP or LVEF did not alter among before, at 6 months, and at 12 months of carnitine administration, but LVMI was increased at 12 months of carnitine administration compared with before carnitine administration. Mean mitral E/A wave velocity ratio did not alter among before, at 6, and at 12 months in the control or carnitine groups (Table 3).

**Table 3:** Changes in plasma B-type natriuretic peptide concentrations and left ventricular function in the control and carnitine group

	Before	6 months	12 months
Control group (*n* = 26)			
Plasma B-type natriuretic peptide, ng/L	291.2 ± 174.6	285.6 ± 165.9	310.1 ± 168.8
Left ventricular ejection fraction, %	52.2 ± 4.8	52.0 ± 5.7	51.9 ± 6.0
Left ventricular mass index, g/m^2^	126.4 ± 24.1	126.3 ± 25.1	126.5± 23.8
Mitral early to atrial (E/A) wave velocity ratio	1.1 ± 0.6	1.1 ± 0.7	1.1 ± 0.6
Carnitine group (*n* = 27)			
Plasma B-type natriuretic peptide, ng/L	249.2 ± 229.1	274.2 ± 213.2	360.2 ± 440.3
Left ventricular ejection fraction, %	53.2 ± 11.9	54.0 ± 11.7	53.7 ± 9.5
Left ventricular mass index, g/m^2^	126.6 ± 24.8	138.0 ± 39.3	140.9 ± 34.8^*^
Mitral early to atrial (E/A) wave velocity ratio	1.2 ± 0.5	1.1 ± 0.6	1.1 ± 0.6

^*^ *P* < 0.01 versus before.

### Carnitine and myocardial fatty acid imaging

**Figure 2:**
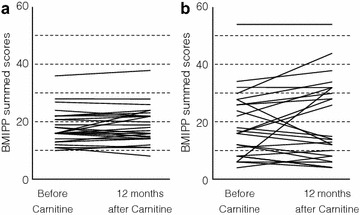
Changes in BMIPP summed scores before and 12 months after carnitine administration. **a** Control group, *n* = 26. **b** Carnitine group, *n* = 27.

**Figure 3:**
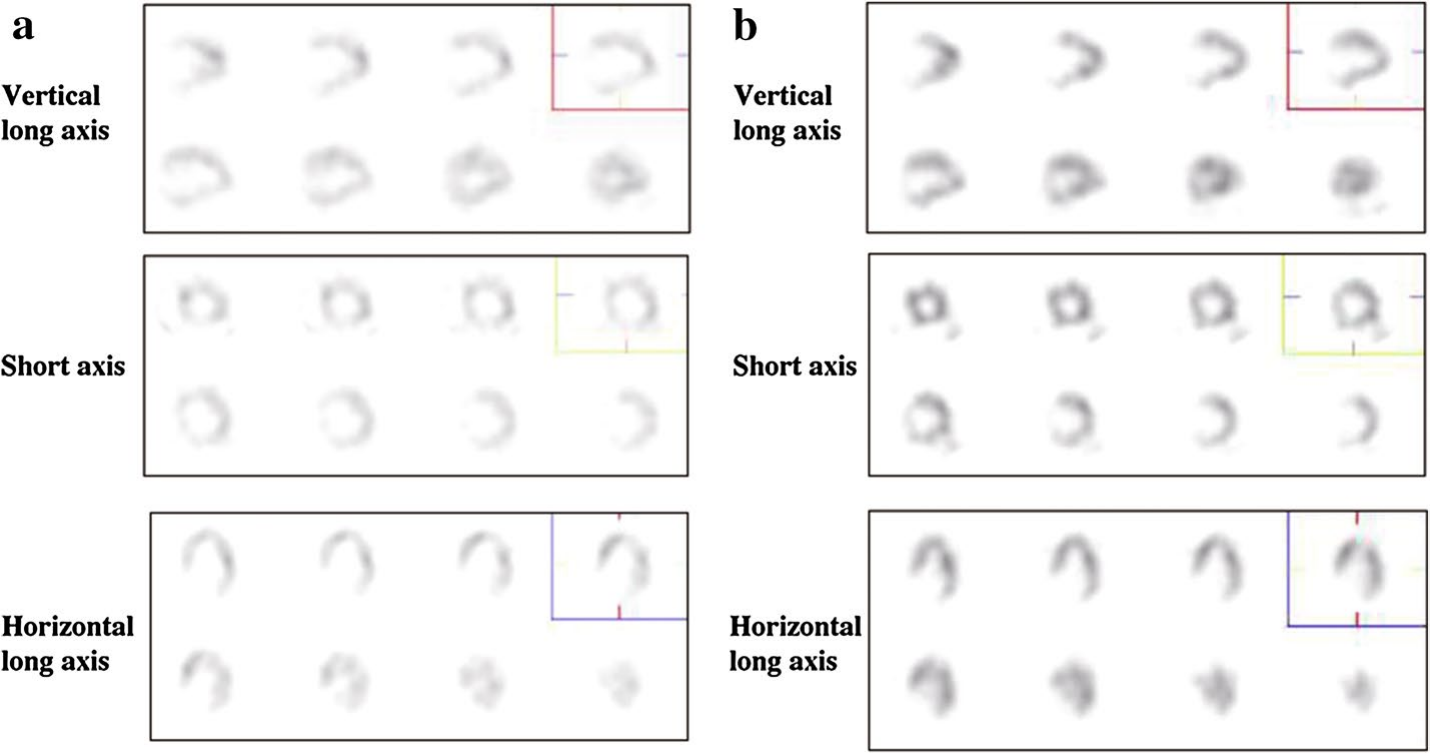
BMIPP SPECT images from a participant who showed improvement after administration of l-carnitine: a 70-year-old non-diabetic woman whose dialysis duration was 22 years. BMIPP summed scores were 28 before administration of l-carnitine (**a**) and 12 after administration of l-carnitine for one year (**b**). Left ventricular ejection fraction evaluated by echocardiography also improved, from 43 to 62% after administration of l-carnitine. Her baseline serum albumin concentration was 34 g/L.

In the control group, the mean BMIPP SS did not differ between before and 12 months after carnitine administration (18.2 ± 6.0 versus 18.7 ± 6.4, *n* = 26) (Figure 2a). Since we have reported that decrease in BMIPP SS <−20% might result in improving cardiac mortality of hemodialysis patients in our recent studies (Nishimura et al. 2014, 2015), we subdivided participants of the carnitine and control groups into the following three subgroups according to the changes in BMIPP SS: improved subgroup, changes in BMIPP SS <−20%; deteriorated subgroup, changes in BMIPP SS >20%; unchanged subgroup, changes in BMIPP SS ± 20%. In the control group (*n* = 26), 2 (7.7%), 4 (15.4%), and 20 (76.9%) participants were allocated to the improved, deteriorated, and unchanged subgroup, respectively. In the carnitine group, the mean BMIPP SS also did not differ between before and 12 months after carnitine administration (18.9 ± 11.3 versus 20.7 ± 13.5, *n* = 27) (Figure 2b). In the carnitine group (*n* = 27), 7 (25.9%), 8 (29.6%), and 12 (44.5%) participants were allocated to the improved, deteriorated, and unchanged subgroup, respectively. Figure 3 shows one of the improved cases in the carnitine group. The allocation to improved subgroup was greater (*P* = 0.025) in the carnitine than in the control group, whereas the allocation to deteriorated subgroup did not differ between the two groups (*P* = 0.2).

### Differences in baseline clinical or laboratory data and cardiac function by changes in BMIPP SS

**Figure 4:**
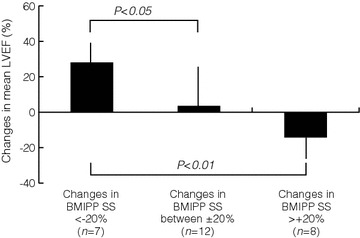
Differences in percent changes in mean left ventricular ejection fraction (LVEF) among subgroups divided by changes in BMIPP summed scores (SS) after intravenous administration of l-carnitine for 1 year.

The baseline mean LVEF was lower in the improved subgroup than in the deteriorated subgroup, and the baseline mean values of serum albumin and total cholesterol concentrations were lower in the improved subgroup than in the unchanged subgroup (Table 4). Mean serum baseline concentrations of total, free, or acyl carnitine and acyl/free carnitine ratio did not differ among the three subgroups (Table 4). In addition, mean serum carnitine concentrations and acyl/free carnitine ratio 1 year after l-carnitine administration did not differ among the subgroups (total carnitine: changes in BMIPP SS <−20%, 464.4 ± 156.5 μmol/L, changes in BMIPP SS ± 20%, 441.3 ± 85.7 μmol/L, changes in BMIPP SS >20%, 420.3 ± 70.5 μmol/L; free carnitine: SS <−20%, 286.4 ± 93.6 μmol/L, SS ± 20%, 255.8 ± 45.9 μmol/L, SS >20%, 259.9 ± 39.7 μmol/L; acyl carnitine: SS<−20%, 178.1 ± 75.0 μmol/L, SS ± 20%, 185.5 ± 45.3 μmol/L, SS >20%, 160.4 ± 37.1 μmol/L; acyl/free carnitine ratio: SS <−20%, 0.62 ± 0.15, SS ± 20%, 0.72 ± 0.12, SS >20%, 0.62 ± 0.11). Mean changes in LVEF after administration of l-carnitine were better in the improved subgroup (28.3 ± 15.9%) compared with the unchanged (3.5 ± 23.7%) or deteriorated subgroup (−13.6 ± 13.1%) (Figure 4).

**Table 4:** Differences in baseline characteristics among the subgroups of carnitine administration

	BMIPP SS changes <-20% (*n*=7)	BMIPP SS changes between ±20% (*n*=12)	BMIPP SS changes >+20% (*n*=8)
Age, y	63.9 ± 11.5	63.3 ± 11.3	64.8 ± 12.0
Male gender, *n* (%)	2 (28.6)	7 (58.3)	5 (62.5)
Dialysis duration, months	143.3 ± 89.1	170.6 ± 112.7	122.0 ± 112.6
Smoking, *n* (%)	1 (14.3)	3 (25.0)	5 (62.5)
Alcohol, *n* (%)	1 (14.3)	3 (25.0)	4 (50.0)
Diabetes mellitus, *n* (%)	4 (57.1)	6 (50.0)	4 (50.0)
Systolic blood pressure before dialysis, mm Hg	135.3 ± 19.4	134.8 ± 19.4	134.0 ± 18.8
Diastolic blood pressure before dialysis, mm Hg	70.1 ± 14.1	68.9 ± 16.1	70.5 ± 14.7
Body mass index, kg/m^2^	21.0 ± 4.5	24.0 ± 4.2	22.9 ± 4.1
Cardiothoracic ratio, %	53.9 ± 5.7	52.0 ± 4.5	54.7 ± 4.7
Left ventricular ejection fraction, %	46.0 ± 8.8†	52.3 ± 14.6	60.9 ± 3.1
Left ventricular mass index, g/m^2^	126.0 ± 35.0	122.4 ± 25.7	133.5 ± 11.0
Mitral early to atrial (E/A) wave velocity ratio	1.1 ± 0.6	1.1 ± 0.7	1.0 ± 0.4
Blood hemoglobin, g/L	104.9 ± 6.5	109.0 ± 10.8	103.0 ± 7.5
Serum albumin, g/L	35.4 ± 2.8*	39.4 ± 2.9	37.9 ± 3.5
Serum calcium, mmol/L	2.2 ± 0.2	2.2 ± 0.1	2.2 ± 0.2
Serum inorganic phosphorus, mmol/L	1.6 ± 0.2	1.5 ± 0.4	1.6 ± 0.3
Serum total cholesterol, mmol/L	3.1 ± 1.0*	4.4 ± 0.9	3.8 ± 0.8
Serum ferritin, pmol/L	403.1 ± 198.2	269.6 ± 253.0	274.1 ± 155.7
Serum intact parathyroid hormone, ng/L	78.1 ± 59.9	122.1 ± 15.9	198.6 ± 96.9
Serum C-reactive protein, mg/L	4.0 ± 3.7	1.3 ± 1.1	2.0 ± 2.6
Plasma B-type natriuretic peptide, ng/L	289.2 ± 245.4	205.6 ± 229.2	279.5 ± 234.1
HOMA-IR, mmol/L・μU/ml	5.8 ± 2.6	5.4 ± 2.4	4.6 ± 2.0
BMIPP summed score	15.1 ± 8.2	22.8 ± 13.2	16.3 ± 9.9
Total carnitine, μmol/L	59.3 ± 52.7	58.9 ± 41.1	62.3 ± 59.0
Free carnitine, μmol/L	36.9 ± 38.6	33.7 ± 23.3	44.1 ± 57.1
Acyl carnitine, μmol/L	22.4 ± 14.4	25.1 ± 18.0	26.2 ± 31.3
Acyl/ free carnitine ratio	0.72 ± 0.15	0.73 ± 0.15	0.64 ± 0.11
Medications			
*α*_1_ blockers, *n* (%)	1 (14.3)	0 (0)	2 (25.0)
*β* blockers, *n* (%)	5 (71.4)	6 (50.0)	6 (75.0)
Calcium blockers, *n* (%)	3 (42.9)	2 (16.7)	2 (25.0)
RAS inhibitors, *n* (%)	3 (42.9)	2 (16.7)	4 (50.0)
Nitrates, *n* (%)	1 (14.3)	2 (16.7)	0 (0)
Antiplatelet drugs, *n* (%)	5 (71.4)	9 (75.0)	4 (50.0)
Anticoagulation drugs, *n* (%)	1 (14.3)	1 (8.3)	3 (37.5)
Statins, *n* (%)	1 (14.3)	4 (33.3)	2 (25.0)

*SS* summed score, *HOMA-IR* the homeostasis model assessment index of insulin resistance, *RAS* renin-angiotensin system.

*P < 0.05 versus the subgroup of BMIPP SS changes between ±20%.

^†^ *P* < 0.05 versus the subgroup of BMIPP SS changes >20%.

### Factors related with improvement in BMIPP SPECT after carnitine administration

In an univariate logistic analysis, changes in BMIPP SS <−20% (Improved BMIPP SPECT) was significantly associated with baseline serum concentrations of albumin, total cholesterol, and CRP, and tended to be associated with baseline LVEF and serum ferritin concentration (Table 5). Mean ERI did not differ between before and after carnitine administration (0.02 ± 0.02 versus 0.02 ± 0.02, *P* = 0.860). Improvement of ERI was found in 10 of 27 patients (37.0%), and aggravation of ERI in 5 of 27 patients (18.5%). Changes in BMIPP SS did not correlate with changes in ERI (*r* = -0.061, *P* = 0.764), and changes in ERI were not associated with decrease in BMIPP SS <−20% (10%: Odds ratio, 0.999; 95% confidence interval, 0.996–1.002; *P* = 0.449). In a multivariate logistic analysis among the factors of *P* < 0.1 in an univariate analysis, decrease in BMIPP SS <−20% was associated with baseline serum albumin concentration (1 g/L: odds ratio, 0.669; 95% confidence interval, 0.456–0.980; *P* = 0.039). In ROC analysis, the cut-off of baseline serum albumin concentration for decrease in BMIPP SS <−20% was 35 g/L (area under the curve: 0.789). Mean serum albumin concentration one year after administration of l-carnitine tended to be higher than that before l-carnitine administration in patients with changes in BMIPP SS <−20% (37.9 ± 1.2 versus 35.4 ± 2.8 g/L, *P* = 0.075, *n* = 7), but did not differ in patients with changes in BMIPP SS ± 20% (38.3 ± 1.8 versus 39.4 ± 2.9 g/L, *P* = 0.162, *n* = 12) or those with changes in BMIPP SS >20% (36.4 ± 3.5 versus 37.9 ± 3.5 g/L, *P* = 0.142, *n* = 8).

**Table 5:** Univariate logistic analysis for BMIPP SS changes <-20%

	Odds ratio	95% CI	*P*
Age (1 y)	0.991	0.921–1.067	0.811
Male gender	0.267	0.041–1.727	0.166
Dialysis duration (1month)	0.999	0.991–1.008	0.862
Smoking	0.250	0.025–2.489	0.237
Alcohol	0.310	0.031–3.111	0.319
Diabetes mellitus	1.333	0.235–7.556	0.745
Systolic blood pressure before dialysis (1 mm Hg)	1.005	0.959–1.053	0.826
Diastolic blood pressure before dialysis (1 mm Hg)	0.998	0.940–1.059	0.942
Body mass index (1 kg/m^2^)	0.824	0.629–1.079	0.159
Cardiothoracic ratio (1%)	1.036	0.864–1.243	0.700
Left ventricular ejection fraction (1%)	0.935	0.867–1.008	0.081
Left ventricular mass index, (1 g/m^2^)	0.999	0.964–1.034	0.938
Mitral early to atrial (E/A) wave velocity ratio (1)	1.289	0.310–5.354	0.727
Blood hemoglobin (1 g/L)	0.801	0.302–2.121	0.655
Serum albumin (1 g/L)	0.669	0.456–0.980	0.039
Serum calcium (1 mmol/L)	0.895	0.002–437.4	0.972
Serum inorganic phosphorus (1 mmol/L)	1.863	0.096–36.204	0.681
Serum total cholesterol (1 mmol/L)	0.244	0.062–0.963	0.044
Serum ferritin (1 pmol/L)	1.004	0.999–1.009	0.097
Serum intact parathyroid hormone (1 ng/L)	0.991	0.981–1.002	0.120
Serum C-reactive protein (1 mg/L)	1.421	1.004–2.010	0.047
Plasma B-type natriuretic peptide (1 ng/L)	1.001	0.997–1,005	0.587
HOMA-IR (1 mmol/L・μU/ml)	1.171	0.790–1.738	0.432
Serum total carnitine (1 μmol/L)	0.999	0.983–1.014	0.870
Serum free carnitine (1 μmol/L)	0.999	0.976–1.023	0.952
Serum acyl carnitine (1 μmol/L)	0.992	0.946–1.040	0.732
Acyl/free carnitine ratio (1)	3.937	0.006–2774.282	0.682
BMIPP summed score (1)	0.952	0.866–1.047	0.311
Medications			
*α*_1_ blockers	1.500	0.115–19.640	0.757
*β* blockers	1.667	0.257–10.792	0.592
Calcium blockers	3.000	0.469–19.176	0.246
RAS inhibitors	1.750	0.296–10.340	0.537
Nitrates	1.500	0.115–19.640	0.757
Antiplatelet drugs	1.346	0.205–8.819	0.757
Anticoagulation drugs	0.667	0.061–7.230	0.739
Statins	0.389	0.038–3.970	0.426

*SS* summed score, *CI* confidence interval, *HOMA-IR* the homeostasis model, *RAS* renin-angiotensin system.

## Discussion

The present study showed that chronic intravenous administration of l-carnitine did not significantly affect mean BMIPP SS evaluated by SPECT in hemodialysis patients with LV dysfunction, although circulating levels of l-carnitine increased almost six-fold at 3 month of administration. When we divided the participants of the carnitine group into the three subgroups according to the increase or decrease in BMIPP SS, 25.9% were allocated to the improved subgroup, whereas 29.6% were allocated to the deteriorated subgroup. Improved uptake of BMIPP in SPECT after l-carnitine administration was associated with betterment of LVEF. Responders and non-responders may exist among hemodialysis patients regarding the effect of l-carnitine on myocardial fatty acid imaging.

Over 70% of the energy required by the normal myocardium under aerobic conditions derives from metabolism of FFA. Under hypoxic or ischemic conditions, FFA metabolism is believed to be suppressed and replaced by glucose metabolism, which requires less oxygen consumption. ^123^I-BMIPP is a branched FFA analogue characterized by resistance to β-oxidation. The metabolism and kinetics of BMIPP in myocardial cells are determined by the following factors: (1) Incorporation from the blood into cardiac muscle cells via the CD36-positive FFA binding protein on the myocardial cell membrane; (2) Back diffusion from myocardial cells into the blood that occurs immediately after incorporation; (3) Intracardiac concentrations of adenosine triphosphate (ATP), which is required for acylation of BMIPP; (4) Accumulation of acyl BMIPP in the lipid pool; and (5) Metabolism to ρ-iodophenyl acetic acid via α- or β-oxidation in mitochondria. Of intracoronary-administered BMIPP in canine myocardium, uptake into myocardial cells was 74%, and retention of acyl BMIPP was 65.3%, whereas metabolism via α- or β-oxidation was only 8.7%. Intracardiac ATP and accumulation in the lipid pool are believed to be significantly associated with early cardiac imaging by BMIPP SPECT (Yamamichi et al. 1995; Tanaka et al. 1997; Kawasaki et al. 1999; Fujibayashi et al. 1990, 1996; Hosokawa et al. 1997).

Experimental administration of etomoxir, a carnitine palmitoyltransferase I inhibitor, to dogs did not affect retention of ^123^I-BMIPP in the heart (Hosokawa et al. 1996). Since only a small fraction of BMIPP is metabolized via α- or β-oxidation in mitochondria as described above, other mechanisms of l-carnitine besides accelerating BMIPP metabolism would be involved in improving fatty acid imaging in hemodialysis patients. Impaired fatty acid metabolism and consequent accumulation of acyl CoA (Coenzyme A) are characteristic of renal failure (Wanner and Hörl 1988). Accumulated acyl CoAs inhibit glucose uptake by disruption of the intracellular signaling cascade that moves the GLUT4 transporter from its intracellular location to the surface of the myocardial membrane (Dresner et al. 1999; Griffin et al. 1999), and also inhibit enzymes important in glucose metabolism such as pyruvate dehydrogenase (Moore et al. 1992; Sugden et al. 1995): accumulated acyl CoAs thereby enhance insulin resistance. In our previous study, impaired fatty acid metabolism evaluated by BMIPP SPECT was in proportion to HOMA-IR in diabetic and nondiabetic hemodialysis patients (Nishimura et al. 2006). This inhibition of acyl CoAs to glucose metabolism is reportedly suppressed by administration of l-carnitine in hemodialysis patients (Günal et al. 1999). l-Carnitine reduces the concentration of acyl CoA esters and improves efflux of excess acyl carnitine from the mitochondria and myocardium via an exchange transport system (Kobayashi and Fujiwara 1994). By reducing acyl CoAs from the mitochondria, l-carnitine may enhance glucose oxidation and increase myocardial synthesis of ATP in spite of impaired fatty acid metabolism.

In this study, baseline serum albumin concentration below 35 g/L was the potent predictor for improvement in BMIPP SPECT by administration of l-carnitine. Serum albumin concentration basically correlates with body protein stores, and serum albumin concentration below 38 g/L suggests a diagnosis of protein-energy wasting (Fouque et al. 2011), which is defined as a pathological state in which there is a continuous decrease or wasting of both protein deposits and energy reserves. Impaired protein anabolism, as well as insulin resistance, is one of the metabolic alterations in patients with end-stage kidney disease (Avesani et al. 2011). In animal studies, l-carnitine administration directly suppressed branched-chain alpha-keto acid dehydrogenase activity; this would lead to increase in intracellular levels of branched-chain amino acids (Owen et al. 2001). Branched-chain amino acids like leucine, valine, and isoleucine play an important role in regulation of body protein turnover (Nakashima et al. 2005; Kimball and Jefferson 2006). Biolo et al. (2008) reported that l-carnitine supplementation was associated with lower rates of leucine oxidation and appearance from proteolysis during the insulin clamp studies than after placebo supplementation. In the present study, mean serum albumin concentration in the subgroup of changes in BMIPP SS <−20% was higher after l-carnitine administration than before, whereas it did not differ between before and after l-carnitine administration in other subgroups. The results of this study indicate that the state of protein-energy wasting may be involved in the mechanism of impaired myocardial fatty acid imaging and that protein-sparing effects of l-carnitine is likely to play a role in improving impaired BMIPP SPECT in this population. Further investigation is needed to clarify this important point.

This study has several limitations. We used CAG to confirm the presence of suspected myocardial ischemia in the participants. “Without significant obstructive CAD” does not necessarily mean that the epicardial coronary arteries are normal. Since histopathological and intravascular ultrasound studies have demonstrated the propensity of angiography to underestimate lesional severity (Porter et al. 1993), we cannot exclude the possibility that some angiographically non-significant lesions were flow-limiting. During the follow-up of 72 participants, 11 participants were lost for follow-up, and 8 participants were discontinued from this study; this high rate of exclusion of participants from the analysis would be a potential source of bias in this study. Since our study was a small, non-blinded, open-labeled trial, it would be difficult to capture a significant effect of l-carnitine and to precisely perform a multivariate analysis. A large, randomized trial is needed to clarify the effects of l-carnitine on impaired myocardial fatty acid imaging.

In conclusion, long-term intravenous administration of l-carnitine did not improve myocardial fatty acid imaging evaluated by BMIPP SPECT in all hemodialysis patients with LV dysfunction. However, improvement in BMIPP SPECT was found in almost one-forth of the participants. Hypoalbuminemia below 35 g/L could be one of the clinical parameters to select hemodialysis patients for administration of l-carnitine to improve fatty acid metabolism and cardiac dysfunction. Intravenous l-carnitine might be an alternative way to treat uremic cardiomyopathy in addition to conventional therapies in a selected group of hemodialysis patients.

## Abbreviations

ATP: adenosine triphosphate
BMIPP: *β*-methyliodophenyl pentadecanoic acid
BNP: B-type natriuretic peptide
CoA: coenzyme A
CAD: coronary artery disease
CAG: coronary angiography
CRP: C-reactive protein
E/A: early to atrial
ESA: erythropoiesis stimulating agent
ERI: erythropoiesis stimulating agent resistance index
FFA: free fatty acids
Hb: hemoglobin
HOMA-IR: homeostasis model assessment index of insulin resistance
LV: left ventricular
LVEF: left ventricular ejection fraction
LVMI: left ventricular mass index
ROC: receiver-operating characteristic
SPECT: single-photon emission computed tomography
SS: summed scores
